# PromarkerD Versus Standard of Care Biochemical Measures for Assessing Future Renal Function Decline in Type 2 Diabetes

**DOI:** 10.3390/diagnostics15060662

**Published:** 2025-03-09

**Authors:** Kirsten E. Peters, Isabella A. Joubert, Scott D. Bringans, Wendy A. Davis, Richard J. Lipscombe, Timothy M. E. Davis

**Affiliations:** 1 Proteomics International, QEII Medical Centre, 6 Verdun Street, Perth, WA 6009, Australia; kirsten@proteomics.com.au (K.E.P.); isabella@proteomics.com.au (I.A.J.); scott@proteomics.com.au (S.D.B.); richard@proteomics.com.au (R.J.L.); 2 Medical School, Fremantle Hospital, University of Western Australia, P.O. Box 480, Fremantle, WA 6959, Australia; wendy.davis@uwa.edu.au

**Keywords:** chronic kidney disease, type 2 diabetes, diabetic nephropathy, standard of care, prognostic risk stratification, plasma biomarker test, proteomics

## Abstract

**Background/Objectives:** The current standard of care for assessing chronic kidney disease complicating diabetes (DKD) includes measurement of estimated glomerular filtration rate (eGFR) and urinary albumin:creatinine ratio (uACR) but both tests have limitations. The present study compared the biomarker-based Promarker^®^D test with conventional biochemical measures for predicting future kidney function decline in adults with type 2 diabetes (T2D). **Methods:** Baseline concentrations of apolipoprotein A-IV, CD5 antigen-like protein and insulin-like growth factor binding protein 3 were combined with age, serum HDL cholesterol and eGFR to generate PromarkerD risk scores for incident DKD/eGFR decline ≥ 30% (the primary endpoint) in 857 adults with T2D (mean age 65.4 years, 54% males). Logistic regression modelling was used to compare the association of (i) PromarkerD, (ii) eGFR, (iii) uACR, and (iv) eGFR plus uACR with this outcome during 4 years of follow-up. **Results:** Study participants were classified by PromarkerD as low (63%), moderate (13%), or high risk (24%) for kidney function decline at baseline. Over a mean 4.2 years, 12.5% developed the primary endpoint. PromarkerD scores showed significantly higher predictive performance (area under the receiver operating characteristic curve (AUC) 0.88 (95% confidence interval (CI) 0.85–0.91)) compared to conventional biochemical measures (AUC = 0.63–0.82). There was a progressive increase in risk with moderate and high risk by PromarkerD exhibiting greater odds of the primary endpoint compared to those at low risk (odds ratios (OR) (95% CI) 8.11 (3.99–16.94) and 21.34 (12.03–40.54), respectively, both *p* < 0.001). **Conclusions:** PromarkerD more accurately identifies adults with T2D at risk of kidney function decline than current usual care biochemical tests.

## 1. Introduction

Chronic kidney disease complicating diabetes (DKD) affects approximately 20–50% of people living with type 2 diabetes (T2D) [1,2,3,4]. It is associated with significant morbidity and mortality, reduced quality of life, extended hospital stays and high healthcare costs, especially in its advanced stages [5,6,7,8]. Early detection and intervention are crucial to reducing the burden of DKD [9,10], especially given that, in addition to conventional measures including optimised blood glucose/blood pressure control and renin–angiotensin system blockade [11,12,13], newer pharmacotherapies, specifically the sodium-glucose cotransporter-2 inhibitors, glucagon-like peptide-1 receptor agonists and mineralocorticoid receptor antagonists, have been shown to reduce DKD progression [14].

The current standard of care for the definition, classification, and prognosis of chronic kidney disease (CKD), defined by the Kidney Disease Improving Global Outcomes (KDIGO) guidelines, comprises measurement of estimated glomerular filtration rate (eGFR) and urinary albumin:creatinine ratio (uACR) [15]. However, both exhibit marked inter- and intra-individual variability, being influenced by factors such as exercise, muscle mass, diet including fluid intake, intercurrent infections and changes in regular medications [14]. Despite acknowledged limitations [16,17], single measures of eGFR and uACR alone and in combination have some prognostic value in predicting major renal events [18]. However, there is a need for better prognostic tests in T2D [18,19].

Promarker^®^D is a validated biomarker-based blood test for DKD risk stratification of people with T2D [18]. It combines the plasma concentrations of three plasma proteins (apolipoprotein A-IV (ApoA4), CD5 antigen-like (CD5L) protein, and insulin-like growth factor binding protein 3 (IGFBP3)) with readily available clinical data (age, serum high-density lipoprotein (HDL) cholesterol and eGFR) to generate individualised scores, categorising patients as low, moderate, or high risk for future adverse renal outcomes. Previous studies have shown that PromarkerD scores accurately predict incident DKD (eGFR < 60 mL/min/1.73 m^2^) as well as eGFR decline ≥ 30% over four years [20,21]. The predictive ability of PromarkerD is equivalent to, or greater than, other biomarker-based prognostic tests for DKD in T2D [20,22,23].

The aim of the present study was to evaluate the performance of PromarkerD compared to eGFR and uACR in predicting future kidney function decline in a community-based cohort of people with T2D.

## 2. Materials and Methods

### 2.1. Participants

Plasma samples and clinical data from participants in the Fremantle Diabetes Study Phase II (FDS2) [24] were used in the present study. We included those FDS2 participants with T2D who attended assessments at baseline and Year 4 between 2008 and 2014, and for whom valid eGFR, uACR and PromarkerD results were available. The FDS2 was approved by the Human Research Ethics Committee of the Southern Metropolitan Area Health Service. All participants gave written informed consent.

### 2.2. Clinical Assessment

Comprehensive sociodemographic and clinical characteristics of all participants were obtained via detailed questionnaires and a standardized physical examination [20]. Fasting biochemical tests were performed in a single nationally accredited laboratory. The Chronic Kidney Disease Epidemiology Collaboration (CKD EPI) equation was used to calculate eGFR [25]. A uACR was determined using a first-morning urine sample. Stored plasma samples were kept at −80 °C until analysis for PromarkerD biomarker assay.

### 2.3. Biomarker Quantification and Promarkerd Scoring

Plasma biomarker concentrations of apolipoprotein A-IV (ApoA4), CD5 antigen-like (CD5L), and insulin-like growth factor binding protein 3 (IGFBP3) were measured by targeted mass spectrometry (multiple reaction monitoring) as previously described [20]. Changes in relative peptide abundances were measured against an ^18^O-labelled reference plasma to give peak area ratios for each biomarker. Using a validated algorithm, the PromarkerD score (from 0 to 100) was derived from the baseline concentrations of three biomarkers and age, serum HDL-cholesterol, and eGFR [20,21]. In addition to absolute individual scores, participants were categorized into three risk groups based on PromarkerD scores as low (<10%), moderate (10% to <20%) and high (≥20%) risk.

### 2.4. KDIGO Risk Stratification

The KDIGO risk categories CKD combine eGFR and uACR to stratify patients by risk of adverse outcomes [15]. These categories range from G1 (eGFR ≥ 90 mL/min/1.73 m^2^) to G5 (eGFR < 15 mL/min/1.73 m^2^), with increasing levels of uACR (A1–A3) indicating higher risk. Microalbuminuria (A2) and macroalbuminuria (A3) were defined as a first-morning uACR ≥ 3 mg/mmol and >30 mg/mmol, respectively. KDIGO risk categories are low (G1–G2 with A1), moderate (G1–G2 with A2; G3a with A1), high (G1–G2 with A3; G3a with A2; G3b with A1), and very high (G3a with A3; G3b with A2–A3; G4 with A1–A3; G5 with A1–A3).

### 2.5. Endpoint Ascertainment

The primary endpoint of this study was a composite measure comprising either (i) incident DKD during the 4-year follow-up in participants with a baseline eGFR ≥ 60 mL/min/1.73 m^2^ or (ii) an eGFR decline of ≥30% (≥7.5%/year) by the 4-year follow-up visit for those with a baseline eGFR < 60 mL/min/1.73 m^2^. Secondary endpoints included the two components separately and, because recent guidelines recommend a greater renal decline as a clinically relevant endpoint [26,27], an eGFR decline of ≥40% (10%/year).

### 2.6. Statistical Analyses

Analyses were conducted in R 4.4.0 with R Studio (v 2024.04.2+764). Logistic regression modelling was used to determine the ability of PromarkerD to predict future kidney decline compared to KDIGO categories, eGFR, uACR and the combination of eGFR and uACR. These models used either continuous or categorical variables without any other independent variables (univariate models). Model performance was assessed using the area under the receiver operating characteristic curve (AUC). The DeLong test [28] was used to compare the AUCs of the PromarkerD model with those for each model incorporating conventional biochemical tests. Model sensitivity (Sn), specificity (Sp), positive predictive value (PPV), and negative predictive value (NPV) were determined at the maximum Youden index (Sensitivity + Specificity − 1) [29] and at moderate and high-risk thresholds (10% and 20%, respectively). Continuous variables (PromarkerD score, eGFR, and uACR) were standardized to Z-scores with a mean of zero and a standard deviation (SD) of 1 for the calculation and comparison of odds ratios between predictive models. Chi-square and Fisher’s exact tests were used to determine differences between the PromarkerD and KDIGO categories in the number (%) of correctly predicted outcomes.

## 3. Results

### 3.1. Participant Characteristics

The present cohort comprised 857 participants (mean age 65.4 years, 54% males; see Table 1) or 54.7% of the FDS2 clinically diagnosed T2D cohort (*n* = 1549). All 857 were assessed at baseline and Year 4, and had valid eGFR, uACR and PromarkerD results at baseline as well as an eGFR at Year 4.

Of those not included in the present analyses, the majority either did not attend the Year 4 assessment (85.3%) or did not have valid PromarkerD biomarker concentrations or other requisite data (14.7%). The majority of the present FDS2 sample (87.5%) did not have DKD at baseline (eGFR ≥ 60 mL/min/1.73 m^2^) and 60.8% were normoalbuminuric (uACR < 3 mg/mmol). Renal decline risk categories are shown in Table 2.

Most participants (56.0%) were categorized as low risk by KDIGO guidelines, 31.5% as moderate risk, 8.1% as high risk, and 4.4% as very high risk. PromarkerD classified 63.2% as low, 13.2% as moderate and 23.6% as high risk for future kidney function decline.

### 3.2. Renal Function Endpoints

At follow-up, a median of 4.2 years (interquartile range (IQR): 4.0–4.4 years) after the baseline assessment, 107 participants (12.5%) had met the primary study endpoint. Of those with an eGFR > 60 mL/min/1.73 m^2^, 9.8% developed DKD during follow-up. In the total sample, 8.1% and 3.4% had eGFR declines ≥30% and ≥40%, respectively.

**Figure 1:**
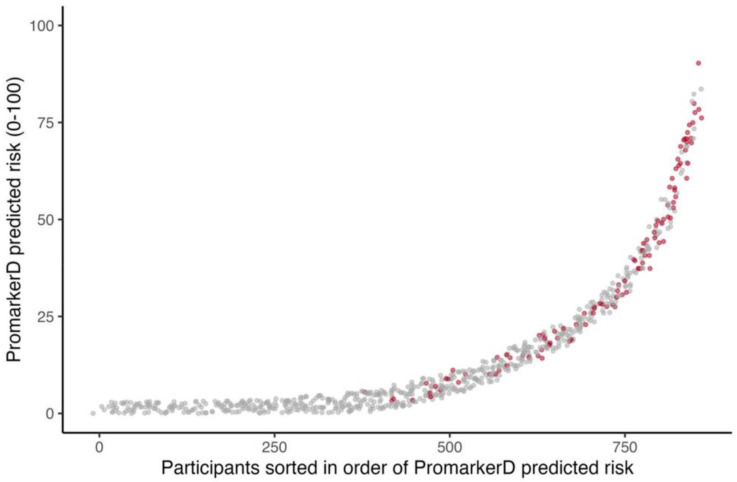
Individual participants ordered by increasing PromarkerD risk score (abscissa) plotted against the magnitude of the score (ordinate). The participants represented by grey dots did not develop the primary endpoint and those represented by red dots did. Jittering is applied to improve the visualization of overlapping points.

PromarkerD risk scores were higher in those who reached the primary endpoint compared with those who did not (see Figure 1), and significantly more participants reached the primary endpoint in the highest-risk (36.1%) and moderate-risk (17.7%) categories compared to the low-risk group (2.6%, both *p* < 0.001). Similarly, significantly more participants in the KDIGO very high-risk group (36.8%) experienced kidney function decline compared to 9.4% in the KDIGO low-risk group (*p* < 0.0001). However, only 13.0% of KDIGO high-risk and 14.4% of moderate-risk group individuals reached the primary endpoint (*p* = 0.458 and 0.046, respectively).

**Figure 2:**
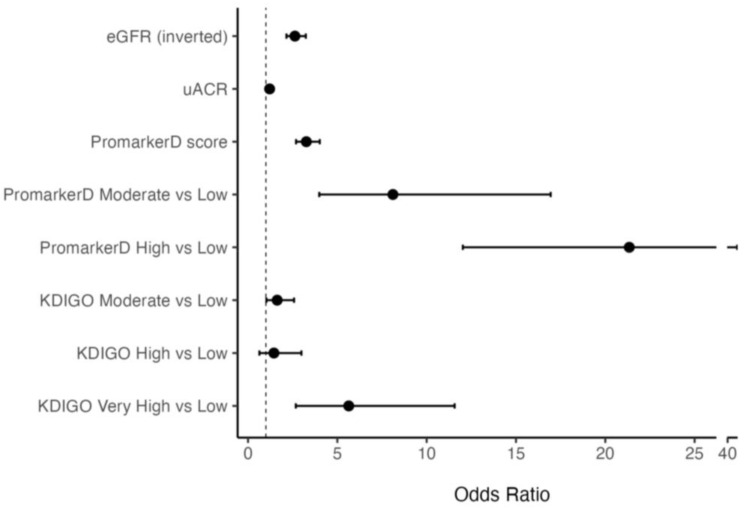
Odds Ratios (ORs) and 95% confidence interval (CI) for models predicting the primary renal outcome. The vertical dashed line at OR = 1 indicates no effect. The bars represent different models, specifically PromarkerD score as a continuous variable and as risk categories, eGFR (inverted for ease of comparison), uACR and KDIGO risk categories.

Higher PromarkerD scores were associated with greater odds of future renal function decline compared to eGFR and uACR (see Figure 2). Specifically, each 1 standard deviation (SD) increase in PromarkerD score was associated with an odds ratio (OR) of 3.26 (95% CI 2.69–4.02). The ORs per 1 SD increase for eGFR and uACR were lower at 2.63 (2.15–3.24) and 1.21 (1.04–1.42), respectively. Participants classified as moderate and high risk by PromarkerD had significantly higher odds of experiencing the primary study endpoint compared to those at low risk (OR 8.11 (3.99–16.94) and 21.34 (12.03–40.54), respectively, both *p* < 0.001). KDIGO risk categories showed variable and more modest degrees of association with the OR for moderate versus low risk at 1.86 (1.18–2.92), high versus low risk at 1.28 (0.51–2.81), and very high versus low risk at 6.70 (3.14–13.99).

### 3.3. Model Performance

**Figure 3:**
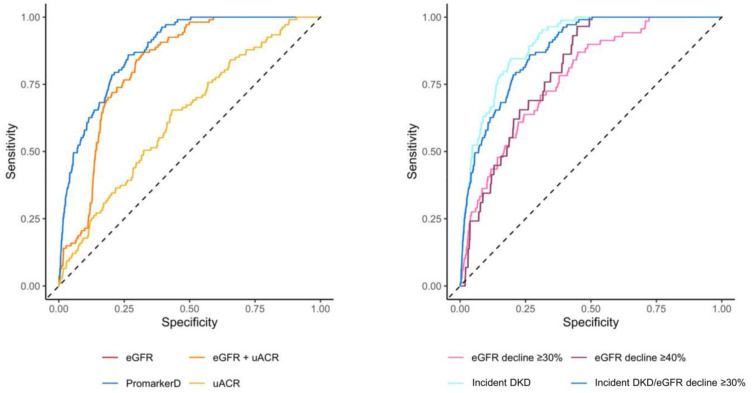
Areas under the curve (AUC) of the receiver operating characteristic for PromarkerD, eGFR, uACR, and eGFR + uACR in predicting the primary renal outcome (left-hand panel) and for PromarkerD in predicting primary and secondary outcomes (right-hand panel). The dashed diagonal line represents the line of no discrimination (AUC = 0.5). In the left-hand panel, the curves for eGFR and eGFR + uACR were colinear and so only the eGFR curve is shown.

PromarkerD demonstrated significantly higher predictive performance compared to conventional tests for predicting kidney function decline (AUC 0.88 (95% CI 0.85–0.91) versus AUCs of 0.63 (0.58–0.68) and 0.82 (0.79–0.85) for uACR and eGFR, respectively, both *p* < 0.001; see Figure 3 and Table 3).

Similarly, PromarkerD showed a better performance than the combination of eGFR and uACR (AUC 0.82 (0.79–0.85), *p* < 0.001). PromarkerD also showed higher specificity and PPV compared to conventional biochemical tests, while maintaining comparable sensitivity and NPV. At the moderate-risk threshold, PromarkerD demonstrated a high NPV (97.2%) and sensitivity (86.1%). High-risk PromarkerD scores were associated with high specificity (82.5%), with a PPV of 37.0%. PromarkerD demonstrated strong predictive power for incident DKD with an AUC of 0.90 (95% CI 0.87–0.93) and sensitivity and specificity of 84.5% and 81.0%, respectively.

For eGFR decline of ≥30% and ≥40% over 4 years, PromarkerD had lower AUC values of 0.78 (95% CI 0.72–0.83) and 0.80 (95% CI 0.74–0.86; see Figure 3). Sensitivities for these outcomes were high at 87.0% and 93.1%, with lower specificities (55.1% and 57.2%).

### 3.4. PromarkerD in KDIGO Low-Risk Participants

There were 480 of the present FDS2 participants who were classified as low risk by KDIGO (eGFR ≥ 60 mL/min/1.73 m^2^ and uACR ≤ 3 mg/mmol). Despite this, 45 (9%) had met the primary study endpoint within 4 years (Table 4).

PromarkerD correctly identified 84% (38/45) of these participants as moderate or high risk. Additionally, PromarkerD accurately classified 79% (342/435) of the participants who did not experience the outcome as low risk, with 98% (342/349) of participants categorized as low risk by PromarkerD not developing the outcome.

### 3.5. Distribution of At-Risk KDIGO Participants

Among the 377 participants classified as ‘at-risk’ by KDIGO (moderate, high and very high risk), 62 (16%) developed incident DKD or experienced an eGFR decline ≥30% over the next four years, while 315 (84%) were false positives (Table 5).

PromarkerD identified 89% (55/62) of the patients who developed the outcome as moderate or high risk, correctly classifying 43 out of the 62 patients as high risk and 12 as moderate risk. Furthermore, PromarkerD accurately classified 59% (186/315) of the participants who did not develop the outcome as low-risk, demonstrating a high NPV of 96% (186/193) for low-risk scores.

## 4. Discussion

In the present community-based cohort of 857 participants with T2D, the PromarkerD test significantly outperformed eGFR and uACR, either alone or in combination, in predicting future decline in kidney function. PromarkerD moderate and high-risk scores were highly prognostic of kidney function decline, and they exhibited a progressive increase in risk while KDIGO risk categories did not (see Figure 2 and Figure 3). The PromarkerD test identified 84% of participants with normal biochemical indices of kidney function at baseline who experienced significant renal function decline over the next four years, participants who would have been missed by KDIGO risk classification. Additionally, PromarkerD accurately classified 78% of patients who did not develop the outcome as low risk and thus had an excellent “rule-out” rate. Taken together, these data support the findings of two independent surveys demonstrating that prognostic tests such as PromarkerD can help inform management decisions, including earlier and more intensive interventions for high-risk patients and avoidance of unnecessary treatments for those at low risk [30,31].

In situations in which only simple metrics of renal function are available, a single eGFR and uACR can provide some useful prognostic information [18,19]. Consistent with past studies, the AUCs in the present analyses were up to 0.82, with eGFR as the strongest predictive variable and uACR providing limited additional value. Serial measures of eGFR may improve the prediction of adverse renal and other clinically important outcomes [32] but, in light of the significant intra-individual variability in eGFR measures over time [14], and given that eGFR screening is typically on an annual basis [33], this approach would not facilitate prompt and effective intervention.

The strong prognostic value of PromarkerD in people with T2D who have a normal eGFR and/or uACR but who progress to adverse renal outcomes is an important finding. There is epidemiological evidence of a graded relationship between a uACR within the normal reference range and the subsequent risk of DKD [34] complicating T2D, but the intra-individual variability in this measure [14,17] confounds its use in an individual case. A single eGFR > 60 mL/min/1.73 m^2^ may represent current or declining hyperfiltration which is a marker of future renal dysfunction [35] but which, in the absence of serial measures, may not be detected. PromarkerD appears to offer a reliable independent test of renal prognosis in these situations.

The present study had limitations. The FDS2 cohort, reflecting the nature of observational studies, can be affected by bias related to study recruitment and retention. Although the majority of people with T2D in the FDS2 were participants in the present study, it is possible that they were relatively healthy compared with those who could have attended the Year 4 review but were not included. The strengths of the present study include its large sample size, and detailed baseline and follow-up assessments.

## 5. Conclusions

PromarkerD significantly outperformed conventional biochemical tests in predicting future kidney function decline in community-based people living with T2D. By identifying people at risk for DKD, PromarkerD can facilitate the early adoption of individualised treatment strategies with the potential for reduced healthcare costs.

## Figures and Tables

**Table 1:** Baseline clinical and laboratory characteristics of the 857 FDS2 participants. Data are %, mean ± SD, median [inter-quartile range] or geometric mean (SD range).

Age (years)	65.4 ± 10.4
Male sex (%)	54.0
Ethnic background (%):	
Anglo-Celt	59.2
Southern European	11.1
Other European	7.9
Asian	4.5
Aboriginal	1.9
Other	15.4
Age at diabetes diagnosis (years)	56.1 ± 11.2
Diabetes duration (years)	7.4 [2.0–15.0]
Body mass index (kg/m^2^)	31.4 ± 6.0
Fasting serum glucose (mmol/L)	7.4 (5.6–9.8)
HbA_1c_ (%)	7.1 ± 1.3
HbA_1c_ (mmol/mol)	54 ± 12
eGFR (mL/min/1.73 m^2^)	81.7 ± 18.6
eGFR categories (%):	
≥90 mL/min/1.73 m^2^	39.7
60–89 mL/min/1.73 m^2^	47.8
45–59 mL/min/1.73 m^2^	7.5
30–44 mL/min/1.73 m^2^	3.9
15–30 mL/min/1.73 m^2^	0.8
<15 mL/min/1.73 m^2^	0.2
Urinary ACR (mg/mmol)	2.2 (1.2–5.7)
Urinary ACR categories (%):	
<3 mg/mmol	60.8
3–30 mg/mmol	34.2
>30 mg/mmol	5.0
Serum total cholesterol (mmol/L)	4.3 ± 1.0
Serum HDL cholesterol (mmol/L)	1.24 ± 0.32
Plasma biomarkers (at peak ratio)	
ApoA4	1.1 (0.5–2.1)
CD5L	1.6 (0.7–3.4)
IGFBP3	1.0 (0.6–1.7)

**Table 2:** Study cohort risk classification at baseline by primary endpoint status. The significance of differences between the KDIGO and PromarkerD risk categories was determined using Chi-square and Fisher’s exact test with the respective low-risk category as reference.

	Number (%) in Risk Category		Number (%) That Reached Primary Endpoint		*p*-Value
Total Cohort	857	100	107	12.5	
PromarkerD risk categories:					
Low	542	63.2	14	2.6	Ref
Moderate	113	13.2	20	17.7	<0.001
High	202	23.6	73	36.1	<0.001
KDIGO risk categories:					
Low	480	56.0	45	9.4	Ref
Moderate	270	31.5	39	14.4	0.046
High	69	8.1	9	13.0	0.458
Very high	38	4.4	14	36.8	<0.001

**Table 3:** Comparison of the PromarkerD and biochemical tests (eGFR and uACR) for predicting the primary study endpoint. Sn, sensitivity; Sp, specificity; AUC, area under the curve; PPV, positive predictive value; NPV, negative predictive value. ^†^ Performance measures based on optimal cutoff defined by Youden index (Sensitivity + Specificity − 1). The test performance of PromarkerD is also provided at the moderate-risk (≥10%) and high-risk (≥20%) test cut-offs which are intended for use in clinical practice. ^‡^ Based on DeLong test, testing the null hypothesis that the difference in AUC between each model and the PromarkerD model is zero.

	Sn (%)	Sp (%)	PPV (%)	NPV (%)	AUC (95% CI)	∆AUC	*p*-Value ^‡^
PromarkerD ^†^	86.0	73.5	31.6	97.3	0.88 (0.85–0.91)	Ref	
Moderate risk	86.1	70.0	29.2	97.2			
High risk	71.3	82.5	37.0	95.2			
eGFR ^†^	86.9	67.7	27.8	97.3	0.82 (0.79–0.85)	−0.057	<0.001
uACR ^†^	65.4	56.8	17.8	92.0	0.63 (0.57–0.68)	−0.249	<0.001
eGFR + uACR ^†^	86.9	67.7	27.8	97.3	0.82 (0.79–0.85)	−0.057	<0.001

**Table 4:** PromarkerD stratification of participants classified as KDIGO low risk. Distribution of participants classified as low risk by KDIGO with normal kidney function (eGFR ≥ 60 mL/min/1.73 m^2^ and uACR ≤ 3 mg/mmol) with number (%) of each category meeting the primary study endpoint. ^†^ The outcome for this subgroup was incident DKD, defined as a reduction in eGFR to <60 mL/min/1.73 m^2^ during follow-up, as all patients had baseline eGFR ≥ 60 mL/min/1.73 m^2^.

	Total	No Outcome	%	Outcome ^†^	%
KDIGO risk category					
Low risk	480	435	91	45	9
PromarkerD classification in this subgroup					
Low risk	349	342	98	7	2
Moderate risk	46	38	83	8	17
High risk	85	55	65	30	35
Total	480	452		45	

**Table 5:** PromarkerD stratification of participants classified as at-risk by KDIGO. Distribution of participants classified as ‘at-risk’ according to KDIGO guidelines with number (%) of participants in each category who experienced the primary study endpoint. ^†^ The outcome for this subgroup was incident DKD (a reduction in eGFR to <60 mL/min/1.73 m^2^) or an eGFR decline ≥30% over four years.

	Total	No Outcome	%	Outcome ^†^	%
KDIGO risk category					
Moderate risk	270	231	86	39	14
High risk	69	60	87	9	13
Very high risk	38	24	63	14	37
PromarkerD classification in this subgroup					
Low risk	193	186	96	7	4
Moderate risk	67	55	82	12	18
High risk	117	74	63	43	37
Total	377	315		62	

## Data Availability

The data that support the findings of this study are available on request from the corresponding author. The data are not publicly available due to privacy, ethical or commercial restrictions.

## Abbreviations

The following abbreviations are used in this manuscript:

DKD	Diabetic kidney disease
eGFR	Estimated glomerular filtration rate
uACR	Urinary albumin:creatinine ratio
T2D	Type 2 diabetes (T2D)
AUC	Area under the receiver operating characteristic curve
CI	Confidence interval
OR	Odds ratio
CKD	Chronic kidney disease
KDIGO	Kidney Disease Improving Global Outcomes
ApoA4	Apolipoprotein A-IV
CDL5L	CD5 antigen-like protein
IGFBP3	Insulin-like growth factor binding protein 3
FDS2	Fremantle Diabetes Study Phase II
Sn	Sensitivity
Sp	Specificity
PPV	Positive predictive value
NPV	Negative predictive value
